# Effects of Transient Electrical Acupuncture Stimulation Combined With Rehabilitation Training on Hemorheology, Neurological Function and BDNF in Patients With Cerebral Infarction

**DOI:** 10.3389/fsurg.2022.839523

**Published:** 2022-02-15

**Authors:** Shuangqin Chen, Jianghua Huang, Xuan Tang, Ting Wang, Yahua Zeng

**Affiliations:** ^1^The First Affiliated Hospital, Department of Neurology, Hengyang Medical School, University of South China, Hengyang, China; ^2^The First Affiliated Hospital, Department of Rehabilitation, Hengyang Medical School, University of South China, Hengyang, China

**Keywords:** instantaneous electrical acupuncture stimulation, rehabilitation training, cerebral infarction, hemorheology, nerve function, BDNF

## Abstract

**Objective:**

To explore the effects of transient electric acupuncture stimulation combined with rehabilitation training on hemorheology, neurological function and brain-derived neurotrophic factor (BDNF) in patients with cerebral infarction (CI).

**Methods:**

A total of 90 patients with CI were admitted to our hospital from March 2019 to March 2021. According to the random number table method, 90 patients were divided into a control group (was treated with transient electrical acupuncture stimulation intervention treatment) and a therapy group (was treated with rehabilitation training on the basis of the control group), with 45 cases in each group. NIHSS score to detect neurological deficit; FMA score to detect motor function recovery; the clinical efficacy of the two groups of patients were compared; blood rheology analyzer to detect whole blood high shear viscosity, whole blood low shear viscosity, platelet aggregation rate and fibrinogen indicators; ELISA detects the content of BDNF in serum.

**Results:**

There was no significant difference in NIHSS score, FMA score, clinical efficacy, hemorheology index, and BDNF content between the two groups of patients before treatment (*P* > 0.05). After treatment, the NIHSS score, whole blood high shear visible, whole blood low shear visible, platelet aggregation rate and fibrinogen index of the two groups were lower than those before treatment, and the FMA score and BDNF content of the two groups were higher than those before treatment, and all the above indicators in the therapy group changed significantly compared with the control group (*P* < 0.05). After treatment, the clinical efficacy of the therapy group was better than that of the control group (*P* < 0.05).

**Conclusion:**

The combination of transient electrical acupuncture stimulation and rehabilitation training can inhibit the blood flow index of patients with CI, improve the nerve function, increase the content of BDNF in the patient's serum, and restore the patient's nerve function.

## Introduction

Cerebral infarction (CI), also known as ischemic stroke, accounts for 65–70% of the total number of strokes in my country, and is an acute and critical illness of cerebrovascular disease. Cerebral infarction is mainly due to cerebral hemodynamic disturbance and local brain tissue is in a state of ischemia and hypoxia, which can cause serious damage to the neurological function of the brain of patients (1). With the improvement of the modern medical level, although vascular interventions and thrombolysis can reperfuse brain tissue to a certain extent, thus effectively reducing the degree of cerebral ischemia and hypoxia, yet the endurance of nerve cells to ischemia and hypoxia is very low. Poor, more than 75% of patients with CI still have different degrees of dysfunction even after the brain tissue is restored to perfusion, which seriously affects the quality of life of the patients. While posing a great threat to the physical and mental health of patients, it also places varying degrees of burden on patients, families and society (2). A large number of studies have shown that the human brain has strong plasticity. Rehabilitation training can increase the expression level of nerve growth factor in patients with CI, stimulate the auxiliary motor cortex, and improve brain function, thereby restoring or partially restoring the control of the brain to the subcortical center and improving the motor function of the limbs (3, 4). Electroacupuncture is an important supplement/alternative treatment for cerebrovascular diseases and is widely used in clinical practice. Electroacupuncture regulates the yin and yang meridians by stimulating acupoints, and achieves the balance between yin and yang by the mutual aid of yin and yang (5). It has been confirmed that electroacupuncture combined with exercise training has a significant synergistic effect in the treatment of postoperative motor dysfunction after CI. Brain-derived neurotrophic factor (BDNF) is one of the most representative members of the nerve growth factor family. It is a basic protein with a relative molecular weight of 12,300, with the highest content in the hippocampus (6). BDNF is closely related to CI. It can promote the survival, development, differentiation and repair of a variety of neurons, promote synaptic plasticity, and participate in the enhancement of long-term memory (7). In this study, combined use of transient electrical acupuncture stimulation combined with rehabilitation training to observe its effects on hemorheology, neurological function and BDNF in patients with CI.

## Materials and Methods

### General Information and Grouping

A total of 90 patients with CI were selected in our hospital from March 2019 to March 2021. According to the random number table method, 90 patients were divided into a control group and a therapy group, with 45 cases in each group. All patients met the relevant diagnostic criteria for CI in our hospital. There was no statistically significant difference between the two groups of patients in general information such as age, course of disease, and pathological location (*P* > 0.05), (Table 1).

**Table 1:** Comparison of general information between the two groups of patients.

**Items**	**Control group** (***n*** **= 45)**	**Therapy group** (***n*** **= 45)**	***t*** **/χ^2^**	***P***
Age (years old)	60.30 ± 5.40	61.22 ± 5.34	0.813	0.419
Course of disease (months)	1.32 ± 0.45	1.29 ± 0.43	0.323	0.747
Male/female (cases)	23/22	21/24	0.178	0.673
Pathological location (cases)			0.184	0.912
Basal ganglia CI	21	23		
Internal capsule infarction	15	14		
Middle CI	9	8		
Basic diseases (cases)			0.239	0.971
Hypertension	17	15		
Diabetes	10	11		
Coronary heart disease	7	8		
Hyperlipidemia	11	11		
Complication (cases)			0.479	0.787
Numbness or paralysis of limbs	24	22		
Aphasia	12	15		
Coughing and dysphagia	9	8		

### Inclusion and Exclusion Criteria

#### Inclusion Criteria

Meeting the definition of acute ischemic stroke as defined by the American Association of Neurological Surgeons (8); Patients with stable vital signs; The age was less than 80 years old; Presence of physical dysfunction; Patients voluntarily participated and signed an informed consent form.

#### Exclusion Criteria

Patients with previous history of mental illness or symptoms of dementia; Patients with neurological deficit symptoms or other diseases, such as hemorrhagic stroke, encephalitis, etc.; Patients with cardiogenic cerebral embolism; Patients with malignant tumors, severe heart, liver and kidney dysfunction, blood system and other diseases; Patients with incomplete medical history data; Acupuncture and other related treatment contraindications.

### Treatment Methods

After admission, medical personnel should give all patients basic treatments such as blood pressure management, brain protection agents, improvement of patients' cerebral blood circulation, and anti-platelet aggregation.

#### Control Group

The patients were given transient electrical acupuncture stimulation intervention treatment. Transient electrical acupuncture stimulation: According to the diagnosis of brain damage, the scalp acupuncture and body acupuncture points were diagnosed. The scalp acupuncture points were the motor area, sensory area, superior temporal gyrus, transverse gyrus and lower gyrus of scalp acupuncture. Acupuncture points for body acupuncture, Lianquan, Tinggong, Yifeng, Fengchi, Jiquan and Neiguan of the paralyzed upper limbs, Shenshu, Huantiao, Fengshi, Weizhong, Sanyinjiao, Taichong, etc. of paralyzed lower limbs. The waveform was density wave, current intensity was 10 (2 mA), and the instrument was SDZ-II Huatuo brand electronic acupuncture instrument and electric acupuncture instrument. Operation method: According to the condition, two groups of scalp acupoints and paralyzed body acupoints were taken, and the current intensity was turned on instantly to 10, within 1~5 seconds, and then quickly returned to zero, and the stimulation was performed sequentially. Each acupuncture point was stimulated 2~3 times, and the patient's expression and tolerance should be closely observed during the stimulation. If dizziness occurs, treatment should be stopped immediately, 4–5 times per week.

#### Therapy Group

The patients were treated with rehabilitation training on the basis of the control group. (1) During the treatment process, medical staff should regularly organize the patient to perform postural changes to ensure smooth blood circulation and avoid pressure injuries and other problems. (2) Medical staff should massage the patient's affected limb to ensure that they could effectively achieve reasonable passive activities, so as to achieve the improvement and optimization of the treatment effect. (3) Trauma rehabilitation training: During the treatment process, medical personnel should guide the patient to perform crossed arms and elevation training to train hip joint control. At the same time, the medical staff should guide the patient with knee flexion and lower limb extension training in prone and supine positions to lay a good foundation for subsequent weight-bearing rehabilitation training. (4) Balance training: Medical staff should actively train the patient's balance ability, including bedside standing and seat balance exercises, to effectively realize the patient's rotation activity and back and forth movement in the correct standing and sitting state. (5) Athletic training: Under the premise of ensuring the patient's safety, guiding the patient to perform appropriate walking or up and down stairs exercises. (6) Occupational therapy: During the treatment process, medical staff should guide the patient to train the functions of the hands and upper limbs, including vertebral body, wiping the table, and basketball control. At the same time, the patient should be instructed to achieve triggering activity of muscles through appropriate neurostimulation techniques. The specific content of the above rehabilitation training was selected according to the actual situation of the patient, 1 h/time, 1 time/d, and the rehabilitation training time was 1 month.

### Observation Indicators

#### Neurological Deficits

The National Institutes of Health Stroke Scale (NIHSS) was used to score the neurological deficits of patients in the two group before and after treatment. The scale included 15 scoring items such as language, consciousness, visual field, movement, and limb ataxia. The NIHSS score ranged from 0 to 45 points; the lower the score, the lighter the neurological deficit.

#### Physical Motor Function

The simplified Fugl-Meyer rating scale (FMA) was used to score the recovery of motor function of hemiplegic limbs before and after treatment in the two group. The scale involved sensation, balance, movement, mobility of limbs and joints, pain and other aspects. The total score was 100 points. The higher the score, the better the motor function.

#### Evaluation of Clinical Efficacy

The clinical efficacy of patients was evaluated according to the evaluation criteria for clinical efficacy of stroke adopted by the Fourth National Cerebrovascular Disease Academic Conference. Get well: After treatment, the symptoms and signs of the nervous system of the patient had basically disappeared, or the NIHSS score was reduced by more than 90%, and the life was completely self-care; markedly effective: the symptoms and signs of the nervous system of the patient were significantly improved after the treatment, or the NIHSS score was reduced by 70–89%, and was able to basically take care of themselves; Effective: After treatment, the patient's nervous system symptoms and signs had improved, or the NIHSS score was reduced by 30–69%, and was able to take care of part of life; Invalid: After treatment, the patient's nervous system symptoms and signs did not improve or even worsened, or died.

#### Hemorheology and BNDF Content Determination

Before and after treatment, 4 mL of cubital venous blood was drawn on an empty stomach in the two groups of patients, centrifuged at 3000 r/min for 10 min, and the serum was separated and stored in a refrigerator at −20°C for examination. The LG-R-20 hemorheology analyzer (Beijing Zhongqin Shidi Scientific Instrument Co., Ltd.) measured the whole blood high shear viscosity, whole blood low shear viscosity, platelet aggregation rate and fibrinogen index before and after treatment in the two groups. ELISA method was used to detect the BDNF content in the serum of the two groups of patients. Kit was purchased from elaid biotech Co., Ltd.

### Statistical Methods

Graphpad prism 8.0 software was used for data analysis, and the measurement data were expressed as *M* ± *SD*. The *t*-test was in accordance with the normal distribution, and the Wilcoxon test was not in accordance with the normal distribution. The measurement data were expressed as % and tested by χ^2^, and *P* < 0.05 indicated that the difference was statistically significant.

## Results

### Comparison of NIHSS Scores Between the Two Groups of Patients Before and After Treatment

**Figure 1:**
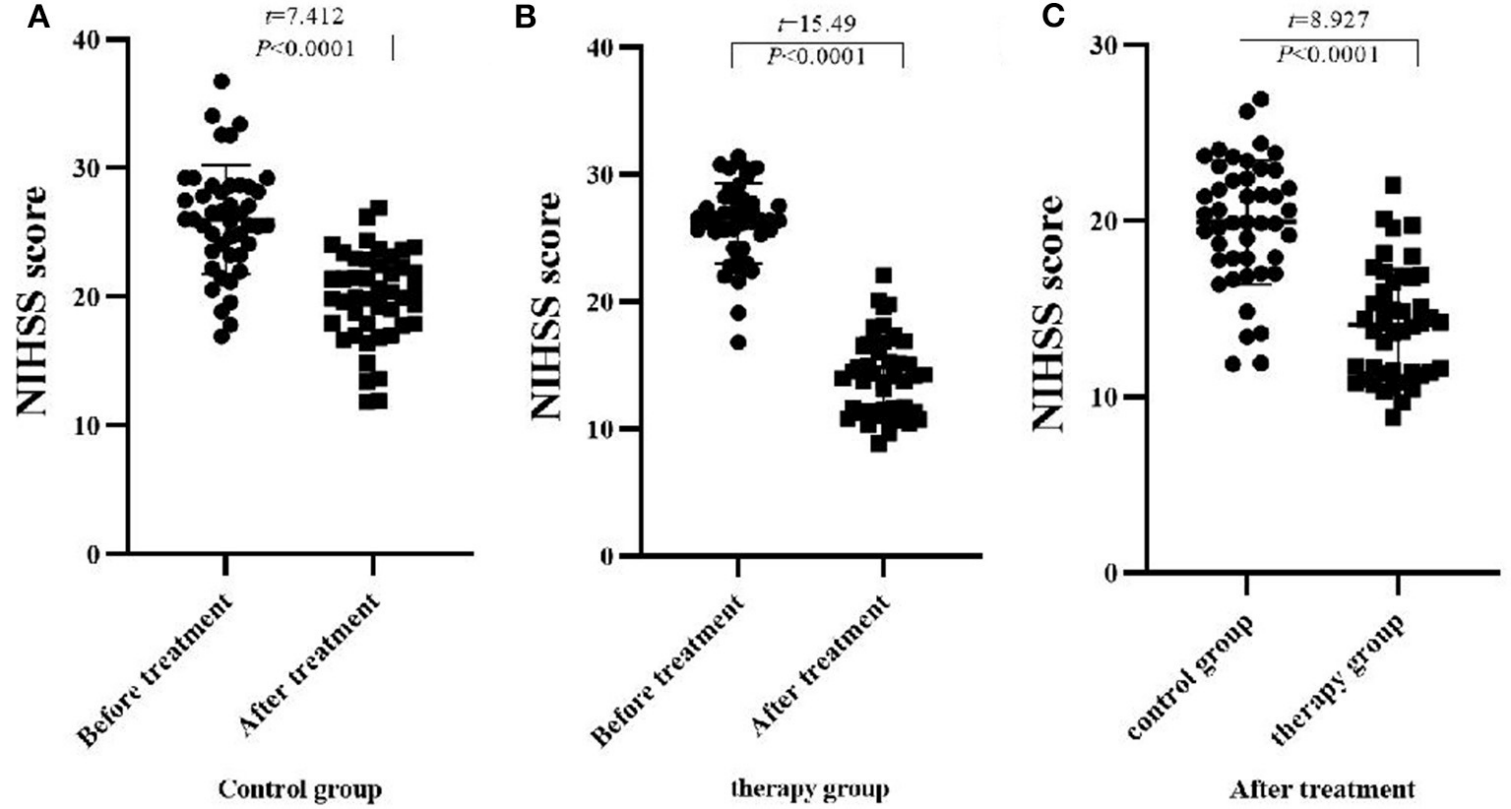
Comparison of NIHSS scores before and after treatment between the two groups (M ± *S*D, scores). **(A)** Comparison of NIHSS scores of the control group before and after treatment; **(B)** Comparison of NIHSS scores of the therapy group before and after treatment; **(C)** Comparison of NIHSS scores of the two groups after treatment.

Before treatment, there was no significant difference in NIHSS scores between the two groups of patients (*P* > 0.05). After treatment, NIHSS scores in both groups decreased significantly, and the therapy group decreased significantly compared with the control group (*P* < 0.05), (Table 2, Figure 1).

**Table 2:** Comparison of NIHSS scores before and after treatment between the two groups (M ± *S*D, scores).

**Group**	***n***	**NIHSS**	
		**Before treatment**	**After treatment**
Control group	45	26.31 ± 4.18	20.46 ± 3.25*
Therapy group	45	26.65 ± 4.27	14.52 ± 3.06^*Δ^
*t*		0.3817	8.927
*P*		0.7036	<0.0001

*Compared with the control group before treatment*,

* *P < 0.05; Compared with the therapy group after treatment*,

Δ *P < 0.05*.

### Comparison of FMA Scores Between the Two Groups of Patients Before and After Treatment

**Figure 2:**
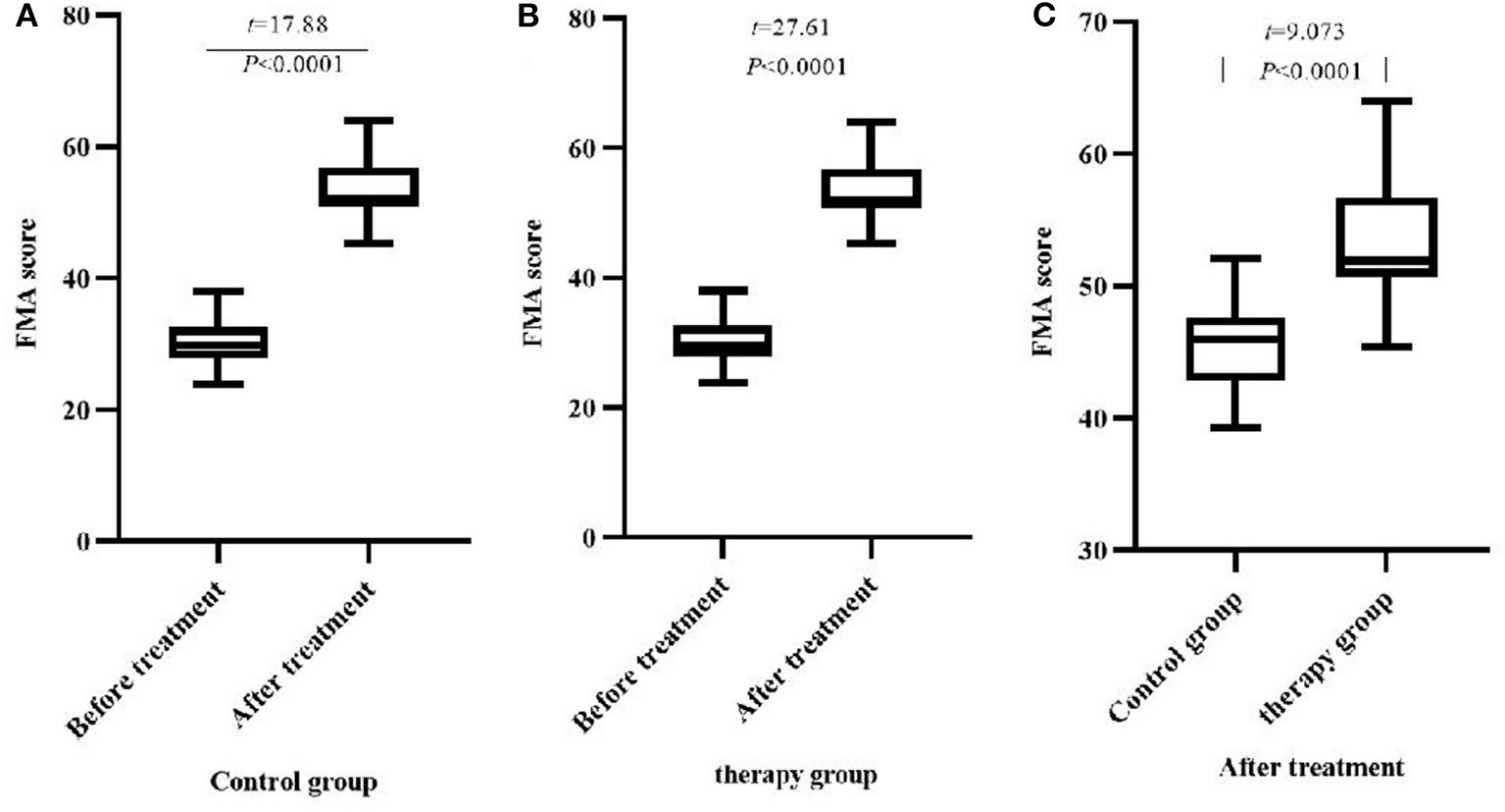
Comparison of FMA scores before and after treatment between the two groups (M ± *S*D, scores). **(A)** Comparison of FMA scores of the control group before and after treatment; **(B)** Comparison of FMA scores of the therapy group before and after treatment; **(C)** Comparison of FMA scores of the two groups after treatment.

There was no significant difference in FMA scores between the two groups before treatment (*P* > 0.05). After treatment, the FMA scores of the two groups increased significantly, and the therapy group increased significantly compared with the control group (*P* < 0.05), (Table 3, Figure 2).

**Table 3:** Comparison of FMA scores before and after treatment between the two groups (M ± *S*D, scores).

**Group**	***n***	**FMA**	
		**Before treatment**	**After treatment**
Control group	45	30.48 ± 3.67	45.27 ± 4.16*
Therapy group	45	30.12 ± 3.25	53.81 ± 4.75^*Δ^
*t*		0.6235	9.073
*P*		0.4926	<0.0001

*Compared with the control group before treatment*,

* *P < 0.05; Compared with the therapy group after treatment*,

Δ *P < 0.05*.

### Comparison of Clinical Efficacy Between the Two Groups of Patients

**Figure 3:**
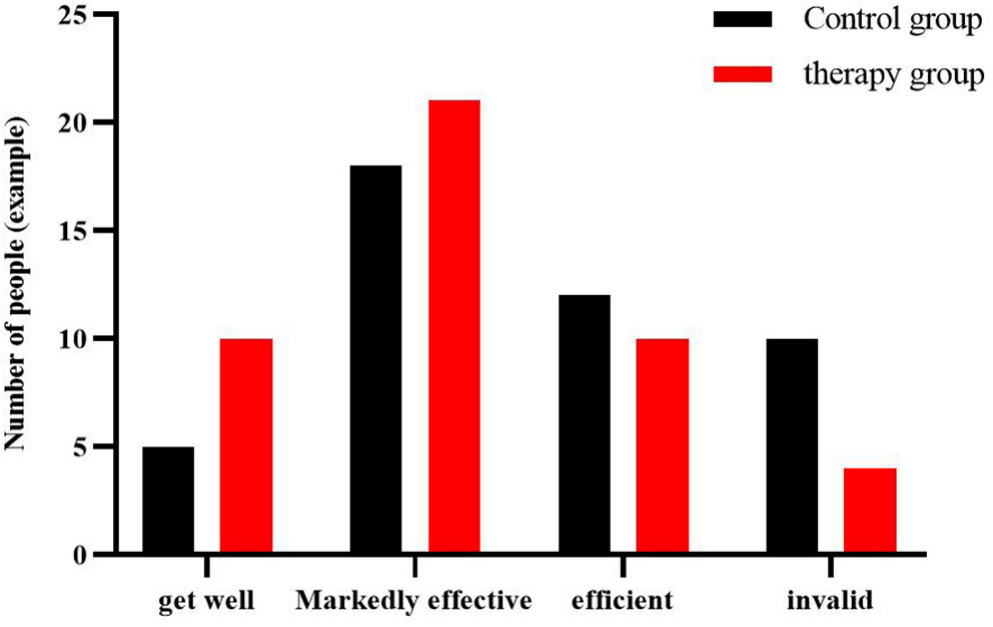
Comparison of clinical efficacy between control group and therapy group (cases).

The total clinical effective rate of patients in the therapy group was 91.1%, and the total clinical effective rate of patients in the control group was 77.8%. Compared with the clinical efficacy between the groups, the treatment group was significantly better than the control group (*P* < 0.05), (Table 4, Figure 3).

**Table 4:** Comparison of clinical efficacy between the two groups of patients [*n* (%)].

**Group**	***N***	**Get well**	**Markedly effective**	**Efficient**	**Invalid**	**Total effective rate**
Control group	45	5	18	12	10	35 (77.8)
Therapy group	45	10	21	10	4	41 (91.1)
*χ^2^*						23.92
*P*						<0.0001

### Comparison of Hemorheology Between the Two Groups of Patients Before and After Treatment

There was no significant difference in hemorheology between the two groups of patients before treatment (*P* > 0.05). After treatment, the whole blood high shear viscosity, whole blood low shear viscosity, platelet aggregation rate, fibrinogen of the two groups were decreased, and the therapy group was significantly lower than that of the control group (*P* < 0.05), (Table 5).

**Table 5:** Comparison of whole blood high shear viscosity, whole blood low shear viscosity, platelet aggregation rate and fibrinogen index before and after treatment in the two groups (M ± *S*D, *n* = 45).

**Group**	**Whole blood high** **shear viscosity (mPa.s)**		**Whole blood low shear** **viscosity (mPa.s)**		**Platelet aggregation** **rate (%)**		**Fibrinogen** **(mg/L)**	
	**Before** **treatment**	**After** **treatment**	**Before** **treatment**	**After** **treatment**	**Before** **treatment**	**After** **treatment**	**Before** **treatment**	**After** **treatment**
Control group	7.42 ± 0.51	6.38 ± 0.46*	15.79 ± 2.61	11.37 ± 0.45*	45.12 ± 2.83	40.24 ± 2.15*	5.01 ± 0.42	3.65 ± 0.37*
Therapy group	7.26 ± 0.48	4.01 ± 0.32^*Δ^	15.93 ± 2.65	7.26 ± 0.32^*Δ^	45.36 ± 2.91	26.47 ± 1.83^*Δ^	4.93 ± 0.38	2.15 ± 0.16^*Δ^
*t*	1.533	28.37	0.2525	49.93	0.3966	32.72	0.9475	24.96
*P*	0.1290	<0.0001	0.8012	<0.0001	0.6926	<0.0001	0.3460	<0.0001

*Compared with the control group before treatment*,

* *P < 0.05; Compared with the therapy group after treatment*,

Δ *P < 0.05*.

### Comparison of Serum BDNF Content Between the Two Groups of Patients Before and After Treatment

**Figure 4:**
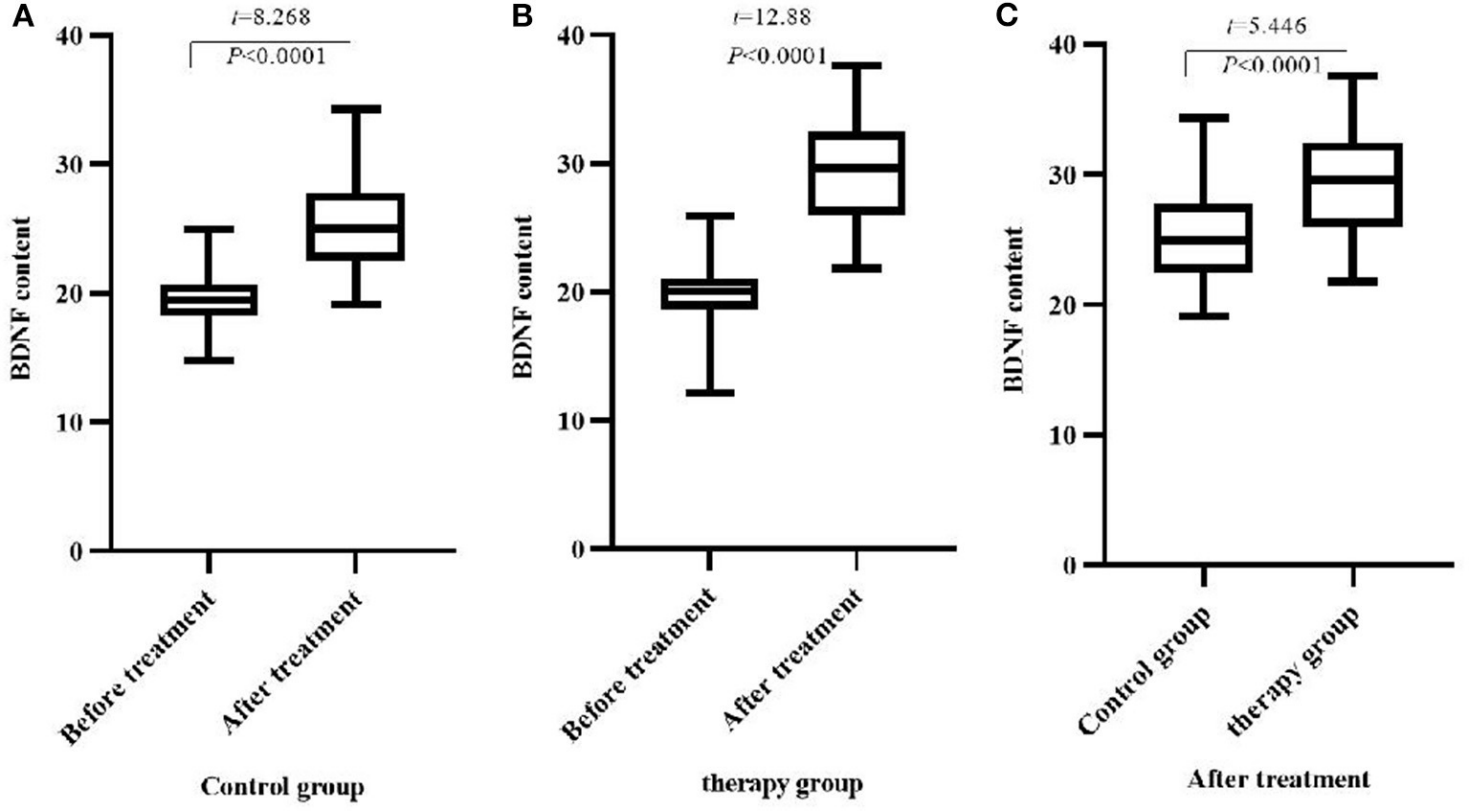
Comparison of BDNF content before and after treatment between the two groups (M ± SD, ng/mL). **(A)** Comparison of BDNF content in the control group before and after treatment; **(B)** Comparison of BDNF content in the therapy group before and after treatment; **(C)** Comparison of BDNF content in the two groups after treatment.

There was no significant difference in serum BDNF content between the two groups before treatment (*P* > 0.05). After treatment, the BDNF content of the two groups increased significantly, and the therapy group increased significantly compared with the control group (*P* < 0.05), (Table 6, Figure 4).

**Table 6:** Comparison of serum BDNF content before and after treatment in the two groups (M ± SD, ng/mL).

**Group**	***n***	**BDNF**	
		**Before treatment**	**After treatment**
Control group	45	19.34 ± 2.16	24.61 ± 3.69*
Therapy group	45	20.08 ± 2.37	29.04 ± 4.02^*Δ^
*t*		1.548	5.446
*P*		0.1252	<0.0001

*Compared with the control group before treatment*,

* *P < 0.05; Compared with the therapy group after treatment*,

Δ *P < 0.05*.

## Discussion

In recent years, the incidence of cardiovascular and cerebrovascular diseases has been increasing year by year. CI is a common critical illness that causes impairment in patients' motor and sensory functions and results in a significant decline in patients' abilities of daily living (9). The current conventional treatment methods for the treatment of CI include inhibiting platelet aggregation, reducing cerebral edema, and restoring blood supply in the infarcted area. However, the rehabilitation effect of patients is often not as expected. It is necessary to further improve the patient's limb exercise ability and self-care ability. Therefore, in this study, combined acupuncture and rehabilitation therapy was given on the basis of conventional Western medicine treatment in the hope of achieving the objective of improving the motor function and self-care ability of patients. The theory of traditional Chinese medicine believes that CI belongs to the category of stroke and dysfunction. The imbalance of kidney, liver, heart, yin and yang and the interaction of qi, fire, wind, blood, and phlegm, which causes disuse of the limbs, blockage of the veins, and dysfunction. The main manifestations are sudden fainting, unconsciousness, hemiparesis, unfavorable language and so on (10). Rescue therapy during the acute phase of CI can help patients to get out of danger, but due to the deficiency of yin in liver and kidney, the patient's qi and blood have not been restored, which can lead to hemiplegia, unfavorable speech and other sequelae. The use of acupuncture to treat CI can significantly reduce neurological damage and help the recovery of motor function (11). Traditional Chinese medicine believes that the governor pulse is the convergence of the three yang meridians of the hand and the three yang meridians of the foot, which can regulate the qi and blood of the yang meridians of the whole body and maintain the vitality of the whole body. Neurological and motor dysfunction in patients with CI is caused by yang deficiency, so by stimulating the head motor area, sensory area, superior temporal gyrus, transverse gyrus, inferior gyrus and various points of the upper and lower limbs, it is beneficial to the the recovery of patient's nerve and motor function, and then improve the prognosis and improve the quality of life of patients. Electrical acupuncture stimulation therapy is one of the important branches of acupuncture in traditional Chinese medicine. Its mechanism is to promote the excitability of central nerve cells and the release of central neurotransmitters by acupuncture scalp and body acupoints and applying a certain amount of current stimulation. It can make the inhibited nerve cells wake up or revive the reversible nerve cells, increase the number of neurons and nerve fibers, and then play a role in promoting nerve reconstruction (12). Relevant studies have shown that the application of instantaneous electrical acupuncture has a significant effect on neurological rehabilitation in patients with acute CI. In addition, the follow-up rehabilitation training is also an important part of the rehabilitation treatment of CI patients. Rehabilitation training can help restore the patient's body, language and brain response ability to the greatest extent. Standardized three-level rehabilitation is a standardized and holistic rehabilitation training model, which uses gradual training content to cooperate with the functional recovery of the injured nerve of the patient, and promotes the recovery of the patient's physical function by maximizing the movement of the affected limb (13, 14).

In this study, the combined treatment of transient electrical acupuncture stimulation and rehabilitation training was given to the patients, and the clinical efficacy of the therapy group was significantly better than that of the control group. In recent years, related reports have pointed out that changes in blood viscosity are an important key factor in ischemic cerebrovascular diseases, and blood viscosity in microcirculation depends on platelet aggregation and fibrinogen (15). This study observes the effects of instantaneous electroacupuncture combined with rehabilitation training on hemorheology, focusing on whole blood high shear viscosity, whole blood low shear viscosity, platelet aggregation rate and fibrinogen as the key observation indicators. The results showed that after treatment, the contents of hemorheological indexes in the two groups were lower than those before treatment, and the contents of hemorheological indexes in the therapy group were lower than those in the control group. This suggests that short-term electroacupuncture combined with rehabilitation training can effectively inhibit platelet aggregation, reduce whole blood viscosity and fibrinogen, so as to inhibit microvascular thrombosis and increase blood oxygen content. Therefore, it plays a good role in improving blood supply and nutrition of brain cells. The results of NIHSS and FMA scores before and after treatment in this study showed that the NIHSS scores of the two groups of patients after treatment were lower than those before treatment, and the FMA scores were higher than before treatment; the NIHSS score of the therapy group was lower than that of the control group, and the FMA score was higher than that of the control group. It can be seen that the combination of transient electrical acupuncture stimulation and rehabilitation training can significantly improve the neurological and motor functions of patients with CI, and improve the physiological quality of patients. It is partially consistent with the results of Sheng et al. (16).

BDNF is a member of the neurotrophic factor family and is mainly distributed in the central nervous system. It plays a very important role in maintaining the survival, growth, differentiation, repair and regeneration of injured neurons. It is essential for maintaining and protecting the survival and growth of neurons (17, 18). And the mouse model shows that the BDNF in the brain tissue can enter the blood through the blood-brain barrier, so it is speculated that the serum level can reflect the BDNF level in the brain (19). The results of this study showed that the serum BDNF content of the two groups of patients after treatment was higher than that before the treatment, and the serum BDNF content of the therapy group was higher than that of the control group. Rising levels of BDNF stimulate and promote the growth and differentiation of neural cells and promote the repair of damaged neurons, and therefore, contribute to the recovery of neurological function. In addition, modern studies have also confirmed that acupuncture can achieve the effect of improving muscle strength by inhibiting neuronal apoptosis, improving cerebral blood circulation, and reestablishing the coordination of regenerating nerves (20).

In summary, the combination of transient electrical acupuncture stimulation and rehabilitation training can inhibit the blood flow index of patients with CI, improve the nerve function, increase the content of BDNF in the patient's serum, and promotes recovery of neurological function in patients.

## Data Availability Statement

The original contributions presented in the study are included in the article/supplementary material, further inquiries can be directed to the corresponding author/s.

## Ethics Statement

The studies involving human participants were reviewed and approved by The First Affiliated Hospital of University of South China. The patients/participants provided their written informed consent to participate in this study.

## Author Contributions

SC was responsible for the writing of the paper. JH and XT were responsible for the design of the research pair and the detection of the results. TW was responsible for the data recording and statistics. YZ was responsible for the guidance of the entire research. All authors contributed to the article and approved the submitted version.

## Funding

This study was supported by subject of Hunan Provincial Health Commission (202214014506).

## Conflict of Interest

The authors declare that the research was conducted in the absence of any commercial or financial relationships that could be construed as a potential conflict of interest.

## Publisher's Note

All claims expressed in this article are solely those of the authors and do not necessarily represent those of their affiliated organizations, or those of the publisher, the editors and the reviewers. Any product that may be evaluated in this article, or claim that may be made by its manufacturer, is not guaranteed or endorsed by the publisher.
